# Supplementary material for: Cyclic bouts of extreme bradycardia counteract the high metabolism of frugivorous bats
Source: eLife. 2017 Sep 19;6:e26686. doi: 10.7554/eLife.26686 (PMC5605195; doi:10.7554/eLife.26686)
Supplement: Supplementary file 1. — N gives the number of observations of heart rate observations used to calculate values. [file elife-26686-supp1.docx]

Table 1. Mean ± sd per bat for heart rates (frequency in beats per minutes, f­_H_) and energy consumption (kilojoules per hour, kJ h^-1^and Watts, W) used during flight, roosting at night, and resting during the day. N gives the number of observations of heart rate observations used to calculate values.

| **bat ID (sex)**  **mass,**  **forearm length** | **day** | **activity** | **n** | **f_H_** | **kJ h^-1^** | **W** |
| --- | --- | --- | --- | --- | --- | --- |
| bat 1 (m)  16.0 g  41.6 mm | 2014-12-04 | flight | 6452 | 786 ± 76 | 4.52 ± 0.95 | 1.25 ± 0.26 |
|  | 2014-12-06 | night activity | 9461 | 580 ± 77 | 2.43 ± 0.64 | 0.68 ± 0.18 |
|  | 2014-12-06 | day roost | 34401 | 565 ± 112 | 2.35 ± 1.09 | 0.65 ± 0.3 |
|  | 2014-12-07 | flight | 5270 | 765 ± 40 | 4.24 ± 0.47 | 1.18 ± 0.13 |
|  | 2014-12-07 | night activity | 18769 | 515 ± 84 | 1.92 ± 0.63 | 0.53 ± 0.17 |
|  | 2014-12-07 | day roost | 37498 | 365 ± 56 | 0.94 ± 0.33 | 0.26 ± 0.09 |
|  | 2014-12-08 | night activity | 17280 | 360 ± 50 | 0.91 ± 0.28 | 0.25 ± 0.08 |
|  | 2014-12-08 | day roost | 24637 | 282 ± 43 | 0.55 ± 0.18 | 0.15 ± 0.05 |
| bat 2 (f)  19.7 g  42.2 mm | 2014-12-08 | flight | 10192 | 756 ± 49 | 5.36 ± 0.74 | 1.49 ± 0.21 |
|  | 2014-12-08 | night activity | 61119 | 502 ± 136 | 2.48 ± 1.28 | 0.69 ± 0.35 |
|  | 2014-12-08 | day roost | 30658 | 297 ± 57 | 0.81 ± 0.36 | 0.22 ± 0.1 |
|  | 2014-12-09 | flight | 1231 | 731 ± 30 | 4.99 ± 0.43 | 1.39 ± 0.12 |
|  | 2014-12-09 | night activity | 9113 | 559 ± 119 | 3 ± 1.16 | 0.83 ± 0.32 |
|  | 2014-12-09 | day roost | 9698 | 324 ± 56 | 0.96 ± 0.39 | 0.27 ± 0.11 |
| bat 3 (m)  17.8 g  42.8 mm | 2014-12-06 | flight | 3554 | 776 ± 60 | 5.01 ± 0.83 | 1.39 ± 0.23 |
|  | 2014-12-06 | night activity | 8688 | 557 ± 76 | 2.56 ± 0.71 | 0.71 ± 0.2 |
|  | 2014-12-06 | day roost | 16850 | 323 ± 43 | 0.83 ± 0.24 | 0.23 ± 0.07 |
|  | 2014-12-07 | flight | 5301 | 765 ± 40 | 4.84 ± 0.53 | 1.34 ± 0.15 |
|  | 2014-12-07 | night activity | 19464 | 512 ± 85 | 2.17 ± 0.72 | 0.6 ± 0.2 |
|  | 2014-12-07 | day roost | 37498 | 365 ± 56 | 1.07 ± 0.37 | 0.3 ± 0.1 |
|  | 2014-12-08 | night activity | 11602 | 316 ± 65 | 0.81 ± 0.35 | 0.23 ± 0.1 |
|  | 2014-12-08 | day roost | 20143 | 310 ± 120 | 0.87 ± 0.96 | 0.24 ± 0.27 |
|  | 2014-12-09 | flight | 618 | 833 ± 78 | 5.8 ± 1.09 | 1.61 ± 0.3 |
|  | 2014-12-09 | night activity | 8883 | 402 ± 107 | 1.38 ± 0.78 | 0.38 ± 0.22 |
|  | 2014-12-09 | day roost | 3465 | 372 ± 167 | 1.33 ± 1.44 | 0.37 ± 0.4 |
| bat 4 (f)  18.8 g  42.2 mm | 2014-12-04 | night activity | 1145 | 409 ± 45 | 1.44 ± 0.38 | 0.4 ± 0.1 |
|  | 2014-12-04 | day roost | 28127 | 405 ± 82 | 1.45 ± 0.64 | 0.4 ± 0.18 |
|  | 2014-12-05 | flight | 3571 | 759 ± 55 | 5.11 ± 0.8 | 1.42 ± 0.22 |
|  | 2014-12-05 | night activity | 15087 | 570 ± 83 | 2.87 ± 0.83 | 0.8 ± 0.23 |
|  | 2014-12-05 | day roost | 39053 | 397 ± 108 | 1.44 ± 0.93 | 0.4 ± 0.26 |
|  | 2014-12-09 | flight | 1231 | 731 ± 30 | 4.71 ± 0.41 | 1.31 ± 0.11 |
|  | 2014-12-09 | night activity | 9231 | 556 ± 121 | 2.81 ± 1.11 | 0.78 ± 0.31 |
|  | 2014-12-09 | day roost | 9580 | 324 ± 56 | 0.9 ± 0.37 | 0.25 ± 0.1 |
|  | 2014-12-10 | flight | 17 | 712 ± 10 | 4.46 ± 0.13 | 1.24 ± 0.03 |
|  | 2014-12-10 | night activity | 919 | 500 ± 98 | 2.24 ± 0.84 | 0.62 ± 0.23 |
|  | 2014-12-10 | day roost | 35155 | 377 ± 94 | 1.28 ± 0.77 | 0.36 ± 0.21 |
