# Supplementary material for: Cyclic bouts of extreme bradycardia counteract the high metabolism of frugivorous bats
Source: eLife. 2017 Sep 19;6:e26686. doi: 10.7554/eLife.26686 (PMC5605195; doi:10.7554/eLife.26686)
Supplement: Supplementary file 2. [file elife-26686-supp2.docx]

Table 1. Individual fitted equations of a 1 pool exponential model for incorporation of ingested δ^13^C into breath carbon dioxide.

1 pool model: δ^13^C breath(*t*) = δ^13^Cbreath(∞) + [δ^13^Cbreath(0) – δ^13^Cbreath(∞)] e^-t/k^

| **Individual** | **δ^13^C_breath_(∞)** | **δ^13^C_breath_(0) – δ^13^C_breath_(∞)** | **k** | **t_50_ (min)** |
| --- | --- | --- | --- | --- |
| A | -9.376 | -18.172 | -0.013 | 51.608 |
| B | -15.267 | -13.308 | -0.100 | 6.942 |
| C | -14.227 | -15.410 | -0.066 | 10.575 |
| D | -18.359 | -12.200 | -0.103 | 6.726 |
| E | -18.864 | -10.647 | -0.108 | 6.401 |
| F | -16.963 | -13.239 | -0.093 | 7.435 |
| G | -16.679 | -12.809 | -0.142 | 4.892 |
| H | -14.304 | -15.111 | -0.057 | 12.087 |
| Full model | -16.575 ±0.918 | -12.841 ± 1.926 | -0.081 ± 0.029 | 8.55 ± 0.71 |
| Full model excluding indiv A | -16.498 ± 0.836 | -13.138 ± 1.861 | -0.091 ± 0.031 | 7.61 ± 0.71 |
